# Moving beyond threshold‐based dichotomous classification to improve the accuracy in classifying non‐responders

**DOI:** 10.14814/phy2.13928

**Published:** 2018-11-22

**Authors:** Jacob T. Bonafiglia, Matthew W. Nelms, Nicholas Preobrazenski, Camille LeBlanc, Lauren Robins, Simo Lu, Alexander Lithopoulos, Jeremy J. Walsh, Brendon J. Gurd

**Affiliations:** ^1^ School of Kinesiology and Health Studies Queen's University Kingston Ontario Canada; ^2^ Healthy Active Living and Obesity Research Group Children's Hospital of Eastern Ontario Research Institute Ottawa Ontario Canada

**Keywords:** Individual responses, interindividual variability, non‐responder, typical error, VO_2_max

## Abstract

We examined maximal oxygen consumption responses following exercise training to demonstrate the limitations associated with threshold‐based dichotomous classification of responders and non‐responders and proposed alternative methods for classification. Specifically, we: 1) calculated individual probabilities of response, and 2) classified individuals using response confidence intervals (CI) and reference points of zero and a smallest worthwhile change of 0.5 METs. Our findings support the use of individual probabilities and individual CIs to improve the accuracy in non‐response classification.

## Introduction

Many exercise training studies have presented a wide range of observed maximal oxygen consumption (*V*O_2_max) responses to structured exercise training at the individual level (Bouchard and Rankinen 2001; Sisson et al. 2009; Ross et al. 2015). These observations have led to a growing interest in characterizing individuals as “responders” or “non‐responders.”

Much of the recent literature has dichotomously classified individuals as either “responders” or “non‐responders” using a pre‐determined threshold (Scharhag‐Rosenberger et al. 2012; Astorino and Schubert 2014; Ross et al. 2015; Montero and Lundby 2017) including two times the typical error (2x TE) (Bouchard et al. 2012; Bonafiglia et al. 2016; Gurd et al. 2016; Raleigh et al. 2016; Alvarez et al. 2017; de Lannoy et al. 2017; Astorino TA et al. 2018). Although 2x TE is a relatively robust threshold for the classification of “responders” (i.e., the true change for observed responses >2× TE above zero is >90% likely to be positive) (Hopkins 2000a), dichotomous classification of “non‐responders” has significant limitations. First, dichotomous classification fails to consider the continuous range of probabilities of a positive response and almost certainly misclassifies individuals as “non‐responders;” and second, 2x TE has typically been applied relative to zero (Bonafiglia et al. 2016; Gurd et al. 2016; Raleigh et al. 2016; Alvarez et al. 2017; de Lannoy et al. 2017; Astorino TA et al. 2018), which fails to consider “non‐response” within the context of meaningful benefit (Swinton et al. 2018).

Individual probabilities of positive (>0) and meaningful (>the smallest worthwhile change; SWC) responses can be calculated (Hopkins 2000b), and a recent review elegantly described a method that quantifies likely positive and meaningful responses using confidence intervals (CI) (Swinton et al. 2018). However, neither of these approaches have been utilized to improve the confidence in the classification of non‐responders. Thus, the purpose of the current study was to extend the work of Hopkins (2000b) and Swinton et al. (2018) to: 1) highlight the limitations of threshold‐based dichotomous classification of non‐responders using individual probabilities of response (Hopkins 2000b), and 2) present conservative methods of classifying individuals as non‐responders using individual CIs and a reference point of both zero and the SWC. The findings from the present study demonstrate that individual probabilities of response and individual CIs are more informative statistical approaches than threshold‐based dichotomous classification. Further, the application of these approaches can improve the accuracy of non‐responder classification for studies in exercise science.

## Materials and Methods

### Experimental design

To address the primary purposes of the present study, we utilized *V*O_2_max data from a recent parallel‐arm exercise study; details of which have been published elsewhere (Preobrazenski et al. 2018). Briefly, 29 healthy, recreationally‐active males were assigned (via minimization (Treasure and MacRae 1998) based on baseline *V*O_2_max) to 4 weeks of structured exercise training (EX, *n* = 14) that was 30 min of cycling at 65% peak work rate four times per week or a no‐prescribed‐exercise control period (CTL, *n* = 15). *V*O_2_max was assessed 1 week preceding (PRE) and ~72 hours following the final training session of the 4‐week intervention (POST). Two incremental step tests with 1‐min (day 1) or 3‐min (day 2, ~24 hours after day 1 test) stages were completed at PRE and POST. PRE and POST *V*O_2_max were determined as the average of the two *V*O_2_max values obtained during the 1‐ and 3‐min stage tests. Collecting repeated measures at each time point (PRE and at POST) is a recommended approach to reduce the influence of measurement error in observed values (Hopkins 2004; Hecksteden et al. 2015). In an attempt to further reduce the impact of measurement error, we followed standardized equipment calibration procedures and asked participants to refrain from ingesting nutritional supplements or exercising 24 h before, and consuming alcohol and caffeine 12 h before all physiological testing.

Although many studies have used secondary criteria (e.g., respiratory exchange ratio >1.15, heart rate ±10 bpm of age‐predicted max, and blood lactate levels >8 mmol/L) to confirm that a maximal VO_2_ is reached during incremental testing, reports have questioned the validity of these criteria as they can occur at a range of submaximal VO_2_ values (Poole et al. 2008). Accordingly, we decided to not use these criteria when measuring *V*O_2_max at PRE and POST. Additionally, we did not include a verification phase in our incremental step tests as there is debate on the utility/necessity of these phases for quantifying *V*O_2_max (Poole and Jones 2017; Green and Askew 2018).

Each participant attended a preliminary screening session where they were briefed on the study, provided informed consent, and had their height and weight recorded. All procedures performed on human participants were submitted and approved by the Health Sciences Human Research Ethics Board at Queen's University (reference number: 6003260) and conformed to the Declaration of Helsinki.

### Secondary analysis

A secondary analysis of the present study was comparing the TE for *V*O_2_max and peak work rate (WR_PEAK_) and examining differences in individual response classification between these variables. WR_PEAK_ was calculated as the highest 30‐second WR period during the 1‐min stage incremental step tests at PRE and POST (Preobrazenski et al. 2018). Given the evidence demonstrating reductions in WR_PEAK_ with stage lengths of 3 min or greater (Bentley et al. 2007), we did not measure WR_PEAK_ from the 3‐min stage tests. Thus, all WR_PEAK_ analysis was derived from a single PRE and a single POST value.

### Statistical analysis

TE for *V*O_2_max and WR_PEAK_ was calculated using the change in these variables from PRE to POST in the CTL group as recently recommended (Williamson et al. 2017). Specifically, the TEs were calculated using the following equation (Hopkins 2000a):

$$TE=\;\frac{SD_{\mathrm{diff}}}{\sqrt{2}}$$

where SD_diff_ is the standard deviation (SD) of the difference scores (POST minus PRE). Although we (Bonafiglia et al. 2016; Gurd et al. 2016; Raleigh et al. 2016; Edgett et al. 2018) and others (Bouchard et al. 2012; Ross et al. 2015; Alvarez et al. 2017; Montero and Lundby 2017) have previously calculated the TE from two baseline tests, we calculated TE from PRE‐POST changes in the CTL group in the present study as this approach captures more sources of variation. Specifically, repeat baseline tests estimates the measurement error (i.e., technical error and day‐to‐day biological variation [Hopkins 2000a]) whereas PRE‐POST changes in CTL estimates measurement error and the within‐subject variability caused by changes in behavioral/environmental factors across an intervention (Williamson et al. 2017).

We calculated a TE of 1.08 mL/kg/min and 13.86 W for *V*O_2_max and WR_PEAK_, respectively. These values resulted in a 2x TE of 2.16 mL/kg/min and 27.71 W for *V*O_2_max and WR_PEAK_, respectively. As recommended by Hopkins (2000a), we compared the TEs across these variables by expressing the TEs as a percentage of the mean of PRE and POST CTL data (herein referred to as the coefficient of variation [CV] for *V*O_2_max and WR_PEAK_).

These TEs were subsequently utilized to calculate individual probabilities of response being greater than zero and the SWC as described by Hopkins (2000b). Fifty percent and 90% CIs of individual responses were calculated as described by Swinton et al. (2018) using a TE multiple that had been adjusted for a sample size of 10. Because the sample size used to calculate TE impacts the certainty in the estimated TE value, adjusting the width of CIs with different multiples is a recommended approach to accommodate studies with sample sizes less than 50 (Swinton et al. 2018).

As recommended by Swinton et al. (2018), we chose an SWC that was expected to be below the expected change for most individuals by a difference greater than our TE. Specifically, for *V*O_2_max we chose a 0.5 MET was chosen because it is likely clinically meaningful (Ross et al. 2016) and fits the criteria outlined by Swinton et al. (2018). Because we are unaware of a clinically meaningful change for WR_PEAK_, we used an SWC of 0.2 times the standard deviation of baseline measures as previously recommended (Hopkins et al. 2009; Swinton et al. 2018).

For our secondary analysis we also calculated the TE for *V*O_2_max using *V*O_2_max values derived from the 1‐min stage tests (1‐MIN) only (i.e., instead of an average from both 1‐MIN and 3‐MIN tests as described above) because WR_PEAK_ was only measured during the 1‐MIN tests. The TE for *V*O_2_max from the 1‐MIN tests was 3.04 mL/kg/min.

## Results

### Limitations of dichotomous classification of responders and non‐responders

**Figure 1:**
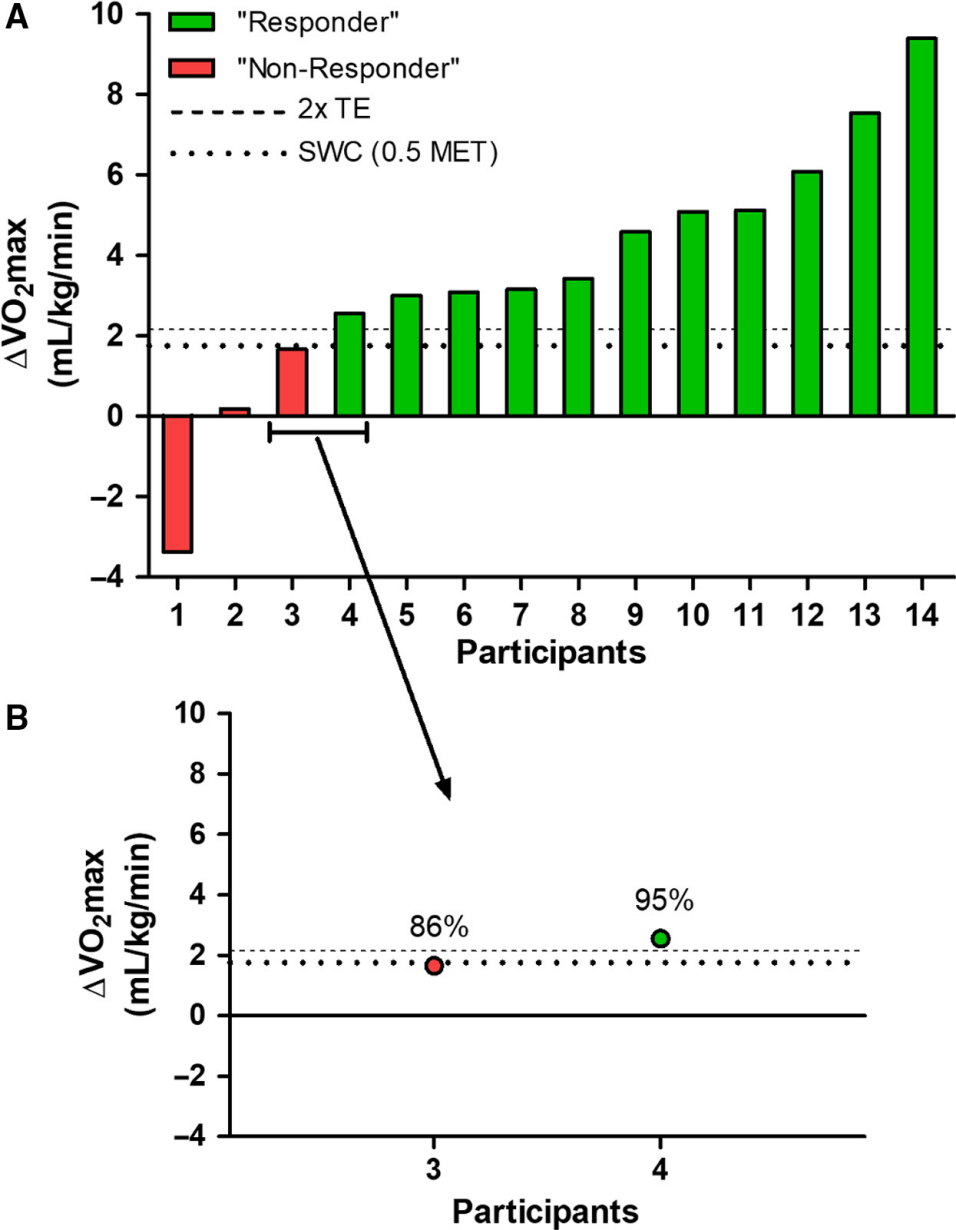
(A) Observed individual VO_2_max responses and 2x TE threshold‐based dichotomous classification of responders and non‐responders. (B) Observed VO_2_max responses and probabilities of a positive response (>0 mL/kg/min) for participants 3–4.

Figure 1 presents the individual observed *V*O_2_max responses to EX. Using a threshold of 2x TE above zero (Fig. 1A), participants were dichotomously classified as “responders” or “non‐responders.” Calculating individual probabilities of response (Fig. 1B) revealed that classifying Participant 4 as a “responder” following the 2x TE approach was appropriate given that there is a 95% chance that this participant had a positive response (i.e., >0 mL/kg/min). Conversely, because there is an 86% chance that Participant 3 had a positive response (Fig. 1B), it is highly likely that classifying this participant as a non‐responder would be a misclassification. This finding demonstrates that the 2x TE approach risks misclassification of non‐responders, particularly for individuals whose observed responses fall just below the dichotomous classification threshold (e.g., Participant 3).

**Figure 2:**
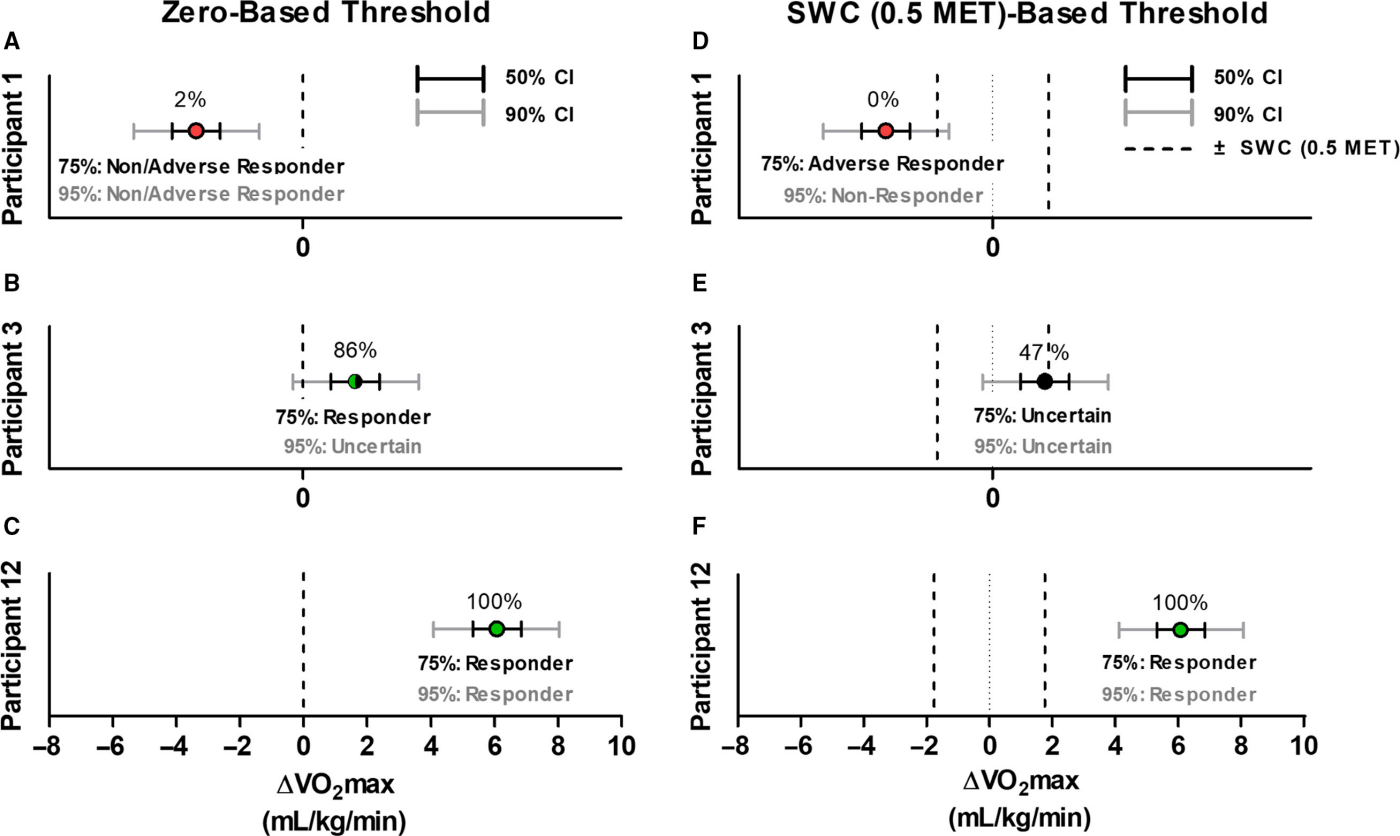
Observed VO_2_max responses, response 50% CIs (black) and 90% CIs (gray), and probabilities of a positive response (percentages) relative to a zero‐based threshold (A–C) and a SWC‐based threshold (D–F) for three representative participants. Participant numbers correspond to Figure 1A. Red, black, and green circles represent non/adverse, uncertain, and positive responders, respectively.

Although Figure 1A presents the probabilities of a positive (>0) response for two representative participants, probabilities of a meaningful positive response (>SWC) can also be calculated (Fig. 2D–F). For a given individual, the probability that their response exceeded the SWC is lower than the probability that their response exceeded zero (Fig. 2).

### Classification of non‐response using response CIs

Figure 2 presents examples of how the recently reviewed response CIs (Swinton et al. 2018) can be used to conservatively classify individual responses using a zero‐based threshold (Fig. 2A–C) and a SWC (Fig. 2D–F) with both 50% and 90% CIs. 50% CIs classifies individuals with 75% certainty while 90% CIs classifies individuals with 95% certainty. Increasing the desired confidence in classifying responses widens the response CI thus increasing the confidence with which individuals can be classified, but also likely increasing the proportion of individuals who cannot be classified with confidence (i.e., classified as uncertain). Using larger CI widths (e.g., 90%) risks making type II errors (i.e., incorrectly classifying individuals as uncertain when they are likely responders or non‐responders), whereas using smaller CI widths (e.g., 50%) risks making type I errors (i.e., incorrectly classifying individuals as responder or non‐responders when they may not be). Because it is not possible to use a single CI width that simultaneously protects against both type I and type II errors, future studies need to decide which type of error they are more willing to risk making when they classify individuals as responders, uncertain, or non‐responders.

When using a zero‐based threshold, individuals can be classified as likely having a positive response (responders; CI lies completely above 0), likely having a negative response (non/adverse responders; CI lies completely below 0) or having an uncertain response (CI overlaps 0). As shown in Figure 2, because participant 12's 90% CI completely lies above zero, they are classified as a “responder” with (at least) 95% confidence (Swinton et al. 2018). Conversely, participant 1 is classified as a “non/adverse responder” with (at least) 95% confidence. Participant 3 is classified as a responder with 75% confidence; however, because their 90% CI crosses 0, they cannot be classified as a responder with 95% confidence and their response would thus be considered “uncertain” at this level of confidence (Swinton et al. 2018).

Using response CIs in conjunction with a SWC‐based threshold allows individuals to be classified as having a meaningful positive response (responders; CI lies completely above the +SWC), a non‐meaningful response (non‐responders; CI lies completely below the +SWC), a meaningful negative response (adverse responders; CI lies completely below the –SWC), or uncertain (CI overlaps the +SWC). Figure 2D–F presents participants 1, 3 and 12 responses classified relative to the SWC (0.5 MET) threshold. Although participant 12 is still classified as a responder with (at least) 95% confidence, participant 1 is classified differently using the 50% and 90% response CI. Specifically, at 75% confidence participant 1 is classified as an adverse responder because their 50% CI completely lies below ‐SWC (Fig. 2D); however, at 95% confidence, this participant is classified as a non‐responder because their 90% response CI crosses the ‐SWC threshold but completely lies below the +SWC threshold (Fig. 2D). Lastly, although participant 3's response falls below the +SWC threshold, this participant cannot be classified as a non‐responder because their CIs overlap the +SWC threshold.

### Comparison of individual *V*O_2_max and WR_PEAK_ responses

WR_PEAK_ had a larger TE than *V*O_2_max when expressing TEs as CVs (WR_PEAK_: 5.29%, *V*O_2_max: 2.39%). However, we calculated a larger CV for *V*O_2_max when using the *V*O_2_max values from the 1‐MIN tests only (6.76%).

Interestingly, despite both *V*O_2_max and WR_PEAK_ being calculated from incremental step tests, some participants were not consistently classified between these variables (Table 1). Specifically, although eight participants were classified as a *V*O_2_max and WR_PEAK_ responder, six participants received a different classification between these two variables (e.g., participant 11 was classified as a *V*O_2_max responder and a WR_PEAK_ non‐responder; Table 1).

**Table 1:** Individual peak oxygen consumption (*V*O_2_max) and work rate (WR_PEAK_) responses classified relative to a smallest worthwhile change (SWC) with 50% confidence intervals for EX participants

Participant	1	2	3	4	5	6	7	8	9	10	11	12	13	14
*V*O_2_max														
WR_PEAK_														

White cells, responder; gray cells, uncertain; black cells, non‐responder. SWC for *V*O_2_max, 0.5 MET (1.75 mL/kg/min); SWC for WR_PEAK_, 0.2 times standard deviation of observed baseline values (10.64 W).

## Discussion

The current study demonstrated how recently proposed statistical approaches (Hopkins 2000b; Swinton et al. 2018) can be used to improve the confidence when classifying non‐responders. The major novel findings are: 1) despite its growing popularity in the individual response literature (Scharhag‐Rosenberger et al. 2012; Astorino and Schubert 2014; Ross et al. 2015; Gurd et al. 2016; Alvarez et al. 2017; de Lannoy et al. 2017; Montero and Lundby 2017; Astorino TA et al. 2018), threshold‐based dichotomous classification approaches misclassify individuals who have a high probability of a positive response as non‐responders, and 2) using response CIs with zero‐ and/or SWC‐based thresholds provides more information than dichotomous classification approaches regarding an individual's response. Application of these statistical approaches has the potential to improve evidence‐informed exercise prescription decision‐making.

### Threshold‐based dichotomous classification overestimates prevalence of non‐response

Despite being a robust approach for classifying responders, threshold‐based dichotomous classification overestimates the prevalence of non‐responders. For example, we recently reported a non‐response rate of 22% (14/63 participants) using a 2x TE threshold for changes in *V*O_2_max following sprint interval training (Gurd et al. 2016). However, reanalysis of this dataset using 90% CIs decreased the non/adverse‐response rate to 6% (4/63 participants) and 10% (6/63 participants) relative to a zero‐based and SWC (0.5 MET)‐based threshold, respectively. Importantly, this re‐analysis suggests that the 2x TE dichotomous classification approach overestimated the prevalence of non‐responders. It is also important to note that utilizing the response CI approach also introduces a group of individuals who cannot be classified as either responders or non‐responders with confidence. Our re‐analysis using 90% CIs resulted in 14% (9/63 participants) and 33% (21/63 participants) of participants being classified as “uncertain” for zero‐ and SWC‐based thresholds, respectively. However, because classifying individuals using response CIs and a zero‐ or SWC‐based threshold increases the confidence with which individuals are classified as non‐responders, the adoption of these statistical approaches should improve the accuracy of future estimates of rates of non‐response.

Although the current work highlights an application of the methods outlined by Swinton et al. (2018), this study is not the first to use CIs to classify individual responses to exercise training. Hecksteden et al. (2018) recently demonstrated an individual classification approach that involves calculating individual response estimates and CIs based on linear regressions of repeated measures collected throughout exercise training. Hecksteden et al. (2018) argue that their proposed approach is superior to threshold‐based dichotomous classification as repeated measures provides a more accurate estimate of an individual's true response. Although our study design did not allow us to adopt Hecksteden et al. (2018) approach (i.e., our study only included pre‐ and post‐training measures), future work should consider including repeated measures to classify individual responses.

### More information for exercise prescription decision‐making

Individual response CIs offer more information than simply dichotomously classifying participants as responders or non‐responders. Using participant 3 as an example, the only information provided by the 2x TE approach is that this participant is classified as a non‐responder (Fig. 1A). Conversely, using the statistical approaches outlined by Hopkins (2000b) and Swinton et al. (2018) shows that participant 3's 50% CI lies above zero with an 86% chance of having a response that exceeded zero (Fig. 2B), suggesting that despite being classified as “uncertain” relative to the SWC (Fig. 2E), this participant likely demonstrated a positive response and possibly (47% chance) demonstrated a clinically meaningful positive response. The added information that can be gathered from these statistical approaches can potentially be used for evidence‐informed exercise prescription decision‐making.

Importantly, evidence‐informed exercise prescription decision‐making requires careful consideration in selecting an SWC threshold. Specifically, determining an SWC can be based on clinical evidence demonstrating the smallest change in a variable that reduces the risk of morbidity/mortality (Hopkins 2018). For variables without clinical evidence, an alternative approach is using an arbitrary SWC of 0.2 times the SD of baseline values (Hopkins et al. 2009; Swinton et al. 2018), as we have done for WR_PEAK_. However, given that numerous studies that have demonstrated the clinical benefits of improvements in *V*O_2_max (Ross et al. 2016), we used a SWC of 0.5 METs instead of the 0.2 times baseline SD approach. Additionally, Swinton et al. (2018) recommend choosing an SWC that is lower than the expected change for most individuals. It is important to note that using a different SWC would shift the threshold(s) used to classify individual responses potentially increasing or decreasing the number of individuals that are classified as responders or non‐responders.

### Comparison of individual *V*O_2_max and WR_PEAK_ responses

Consistent with the findings of a previous report (Montero and Lundby 2017), our CV analysis revealed that the TE for WR_PEAK_ was greater than *V*O_2_max when *V*O_2_max values were taken from the 1‐MIN tests only. However, when the TE for *V*O_2_max was calculated using repeated measures at PRE and POST (i.e., both 1‐MIN and 3‐MIN tests), the TE for *V*O_2_max was lower than the TE for WR_PEAK_ and the TE for *V*O_2_max from the 1‐MIN tests only. The finding that using *V*O_2_max measures derived from two tests at PRE and POST lowered the TE demonstrates the benefit of collecting repeated measures at each time point. Specifically, because collecting repeated measures reduces the impact of measurement error in observed values (Hopkins 2004; Hecksteden et al. 2015), repeated measures may also reduce the magnitude of measurement error in TE estimates. Unfortunately, our study design prohibited us from collecting repeated measures of WR_PEAK_ at each time point and future work is needed to determine whether collecting repeated WR_PEAK_ measures results in a smaller CV than the value reported in the present study.

In addition to differences in TEs, we found that some participants were not consistently classified across *V*O_2_max and WR_PEAK_ responses (Table 1). Although this finding is somewhat surprising given that both *V*O_2_max and WR_PEAK_ are measured during incremental step tests, the observation that participants do not respond similarly across variables is consistent with previous demonstrations of individual patterns of response (Vollaard et al. 2009; Scharhag‐Rosenberger et al. 2012; Astorino and Schubert 2014; Bonafiglia et al. 2016; Gurd et al. 2016; Raleigh et al. 2018).

### Limitations

Although the current study demonstrates that individual probabilities of response and individual CIs are more accurate and informative than threshold‐based dichotomous classification, there are several limitations associated with these statistical approaches. Firstly, the calculation of individual CIs is dependent on the certainty of the TE estimate and assumes that the effect of TE on observed measures is random (i.e., repeated measures normally distribute around the true value). Using large sample sizes to calculate TE may help alleviate the impact of these assumptions/limitations and TE multiples can be used to calculate individual CIs when small sample sizes (<50) have been used to estimate the TE (Swinton et al. 2018), as we have done in the current study. Further, individual probabilities of response are calculated using the TE (Hopkins 2000b) and thus are also influenced by the uncertainty associated with TE estimates derived from small sample sizes. Therefore, a limitation associated with calculating individual probabilities is the lack of a recommended approach to make adjustments when small sample sizes have been used to estimate the TE. Secondly, while we repeated *V*O_2_max measures at pre‐ and post‐training in an attempt to account for the influence of TE in observed measures (Hopkins 2004), it has been recently argued that taking repeated measures throughout the course of an intervention better accounts for TE when classifying individual responses as it estimates the intra‐individual variation in observed measurements (Hecksteden et al. 2018). Lastly, although recent reviews have highlighted the necessity to attribute individual responses to an effect of exercise training *per se* (Atkinson and Batterham 2015; Williamson et al. 2017), the approaches used in the present study simply characterize whether or not individuals have positively responded (or not) to a given intervention and are not designed to determine the cause of each individual's response.

It is important to note that the statistical approaches used in this study represent an application of magnitude‐based inferences (MBI). Unlike traditional null‐hypothesis testing, MBI appraises effect sizes relative to pre‐determined thresholds to gauge whether a given treatment should be implemented (Hopkins and Batterham 2016). Although the statistical principles underlying MBI have been heavily debated (Hopkins and Batterham 2018; Sainani 2018), this debate has focused on performing MBI for group‐level analysis. To our knowledge, all approaches that have been used to classify individual responses involve MBI and it has been argued that MBI is critical for monitoring an individual's progress to exercise training (Buchheit 2018). At present, statistical approaches for classification of individual response that do not rely on MBI are lacking and this represents an important area for future research.

## Conclusion

Our findings support the application of Hopkins (2000b) and Swinton et al. (2018) statistical approaches to more accurately characterize individual responses and classify non‐response. Importantly, our findings suggest that future work should not use threshold‐based dichotomous approaches to classify responders/non‐responders as the field of exercise science moves toward more precisely characterizing rates of non‐response to exercise intervention and prescribing exercise as a personalized medicine. The approaches presented in this study extend beyond *V*O_2_max responses to a short‐term exercise protocol and have utility for characterizing individual responses across a host of other variables and interventions.

## Conflict of Interest

The authors have declared that no conflicts of interests exist.

## Data Accessibility Statement

The raw data supporting the conclusions of this manuscript will be made available by the authors, without undue reservation, upon request.
